# Variability in Key Physiological Parameters in Neurocritical Stroke Patients: A Multicenter Observational Study

**DOI:** 10.3390/jcm15072674

**Published:** 2026-04-01

**Authors:** Omar Alhaj Omar, Patrick Schramm, Tobias Frühwald, Stefan T. Gerner, Kilian Froehlich, Tobias Braun, Martin Juenemann, Heidrun H. Kraemer, Hagen B. Huttner, Anne Mrochen, IGNITE Study Group

**Affiliations:** 1 Department of Neurology, University Hospital Giessen and Marburg, Justus-Liebig-University, Klinikstrasse 33, 35392 Giessen, Germany; patrick.schramm@ukdd.de (P.S.); tobias.fruehwald@neuro.med.uni-giessen.de (T.F.); tobias.braun@lahn-dill-kliniken.de (T.B.); heidrun.kraemer@neuro.med.uni-giessen.de (H.H.K.); anne.mrochen@neuro.med.uni-giessen.de (A.M.); 2 Department of Neurology, University Hospital Carl Gustav Carus Dresden, 01307 Dresden, Germany; 3 Department of Neurology, University Hospital Erlangen, Friedrich-Alexander University, 91054 Erlangen, Germany; stefan.gerner@uk-erlangen.de (S.T.G.); froehlich_k@icloud.com (K.F.); 4 Department of Neurology, Lahn-Dill-Kliniken Wetzlar, 35578 Wetzlar, Germany

**Keywords:** variability, monitoring, intensive care, stroke

## Abstract

**Background**: Effective management of key physiological parameters, such as blood pressure, temperature, blood glucose, and gas exchange, is central to neurocritical care. However, the clinical impact of variability within guideline target ranges after an acute ischemic stroke, intracerebral hemorrhage, or subarachnoid hemorrhage remains unclear. **Methods**: In this multicenter observational study of nine German neurocritical care units, we analyzed in-range measurements over 96 h. Of 524 screened patients, 281 met the predefined criteria for sufficient in-range data. Variability in systolic blood pressure, mean arterial pressure, body temperature, blood glucose, partial arterial pressure of oxygen and carbon dioxide was quantified using the coefficient of variation. Associations between in-range variability of each physiological parameter and clinical outcomes including duration of mechanical ventilation, NIHSS score at discharge, and in-hospital mortality were evaluated using multivariable regression models. **Results**: Variability for all parameters peaked in the first 24 h and then remained largely stable; blood glucose showed a secondary rise after ~60 h. Greater in-range blood glucose variability was associated with in-hospital mortality in hemorrhagic stroke (adjusted OR 1.08; 95% CI 1.00–1.17; *p* = 0.04), while no other parameter’s variability was associated with the evaluated outcomes. **Conclusions**: Overall, in-range variability had limited short-term prognostic value, supporting current guideline-based management.

## 1. Introduction

The management of fundamental physiological parameters including blood pressure, body temperature, blood glucose levels, and parameters of gas exchange remains a major challenge in the treatment of patients with acute neurovascular diseases such as acute ischemic stroke (AIS), intracerebral hemorrhage (ICH), and subarachnoid hemorrhage (SAH) in the neurocritical care setting.

Clinical guidelines endorse adherence to specific target ranges for these parameters, based on varying levels of evidence [1,2,3,4]. However, these recommendations primarily focus on achieving fixed threshold values. Although systematic data on the extent of variability within these recommended ranges are lacking, clinical experience suggests that substantial fluctuations within the recommended ranges are common, particularly during the acute phase of neurovascular diseases. These fluctuations may be driven by impaired or evolving autoregulatory mechanisms and therapeutic interventions such as vasopressor use and mechanical ventilation [5,6].

Beyond absolute threshold violations, variability of physiological parameters within guideline-recommended ranges may itself be clinically relevant in acute cerebrovascular disease. Fluctuations in blood pressure, temperature, blood glucose, and gas exchange parameters such as PaO_2_ and PaCO_2_ may reflect impaired cerebral autoregulation, evolving brain injury, systemic stress responses, and the effects of intensive care interventions including vasoactive therapy, sedation, and mechanical ventilation. Even when mean values remain within recommended targets, repeated oscillations in these parameters could contribute to secondary brain injury by affecting cerebral perfusion, metabolic demand, oxygen delivery, and carbon dioxide-mediated cerebrovascular tone. A better understanding of such in-range variability may therefore provide additional insight into early pathophysiology and its potential association with clinically relevant outcomes in neurocritical care patients.

While short-term blood pressure variability has been associated with unfavorable outcomes in selected ischemic stroke cohorts, the extent and clinical relevance of long-term variability within guideline-recommended target ranges across key physiological parameters in acute neurovascular diseases remain unknown [7,8].

The present multicenter observational study conducted across nine dedicated neurocritical care units in Germany aimed to (i) characterize the temporal dynamics of in-range variability in physiological parameters including blood pressure, glucose levels, body temperature, and ventilation metrics and (ii) evaluate the association of in-range variability with clinical outcomes in mechanically ventilated neurointensive care patients with AIS, ICH, or SAH.

## 2. Materials and Methods

### 2.1. Study Design and Population

We conducted a retrospective, observational, multicenter study involving stroke patients requiring neurointensive care and mechanical ventilation. The study included patients treated at nine dedicated neurocritical care units (NICUs) within tertiary university hospitals in Germany, spanning from 1 January to 31 December 2021. As previously described, patients meeting the following inclusion criteria were enrolled: (i) age > 18 years, (ii) diagnosis of acute neurovascular disease, including AIS, ICH, or SAH (based on International Classification of Diseases, 10th Revision [ICD-10]: I60.x, I61.x, I62.x, I63.x), (iii) admission to the NICU due to intubation and mechanical ventilation, and (iv) a minimum NICU stay of 4 days [9].

Patients were excluded if they had: (i) initial do-not-treat (DNT) or do-not-resuscitate (DNR) orders, (ii) death occurring within 24 h of admission, or (iii) missing data documentation exceeding 33% or incomplete 4 h measurements during the first 96 h of treatment as described [9]. Furthermore, inclusion required that >70% of all recorded physiological parameter values be within guideline-recommended ranges over the 96 h period [9]. Institutional review boards and local ethics committees at all participating sites approved the study, based on the central ethical approval from Giessen University (AZ 177122).

### 2.2. Physiological Parameters and Variability

All clinical and demographic data were extracted from institutional databases. Physiological parameters were recorded upon admission and subsequently at 4 h intervals during the first 96 h after admission. The following parameters were analyzed: (i) systolic blood pressure (SBP) and mean arterial blood pressure (MAP), (ii) body temperature, (iii) blood glucose, and (iv) partial arterial O_2_ and CO_2_ (PaO_2_ and PaCO_2_).

Predefined target ranges were applied according to stroke subtype and clinical context, based on established international guidelines and consensus recommendations, as previously described [9]. In patients with acute cerebral ischemia, the systolic blood pressure target was set between 120 mmHg and 180 mmHg [10]. Following successful mechanical thrombectomy with reperfusion defined as TICI2b, TICI2c, and TICI3, the SBP goal was lowered to 110–160 mmHg [3]. If reperfusion was unsuccessful, SBP levels of up to 180 mmHg were accepted during the first 24 h [11]. In parallel, predefined targets included a MAP of 60–90 mmHg, a body temperature below 37.5 °C, blood glucose levels between 80 and 180 mg/dL, PaO_2_ between 80 and 120 mmHg and PaCO_2_ between 35 and 45 mmHg [3,10,12,13,14,15].

Target systolic blood pressure ranges were 110–140 mmHg for intracerebral hemorrhage and 110–180 mmHg for subarachnoid hemorrhage [1,2,3,16]. Other targets included a MAP of 60–90 mmHg, a body temperature of less than 37.5 °C and blood glucose levels of 80–180 mg/dL [17,18]. Arterial blood gas parameters were managed in accordance with standard neurocritical practice (PaO_2_ 80–120 mmHg; PaCO_2_ 35–45 mmHg) [12,13]. In this cohort, the initial post-admission SBP was classified as non-adherent if it decreased by >90 mmHg compared with the SBP recorded on admission [3]. For each physiological parameter, the coefficient of variation (CV) was calculated separately within the respective predefined time interval as CV = (standard deviation/mean) × 100 [7,19,20]. We chose the CV because it expresses variability relative to the mean and therefore permits comparison across physiological parameters with different units and absolute ranges. This was considered particularly suitable for our retrospective multicenter dataset, whereas absolute measures such as standard deviation are less informative for cross-parameter comparisons.

### 2.3. Statistics

All statistical analyses were performed using JASP (version 0.18.3.0), R (R Foundation for Statistical Computing, version 4.3.3) and BioRender (Mrochen, A. (2026) https://BioRender.com/ckn9efn, accessed on 27 February 2026). Statistical significance was defined as a two-sided α of 0.05. Continuously monitored variables (including arterial-line systolic blood pressure, mean arterial pressure and temperature) were recorded as discrete values at least once every 4 h. Discontinuously obtained measurements (e.g., arterial blood gases and blood glucose) were assigned to the nearest four-hour time point as reported previously [9].

Continuous data were reported as the mean ± standard deviation (SD) if normally distributed, or as the median [interquartile range (IQR)] if not normally distributed. Categorical variables were presented as counts (percentages). The associations between variability of parameters (expressed as the coefficient of variation) and outcome measures were examined using multivariable logistic regression, adjusted for the National Institutes of Health Stroke Scale (NIHSS) at admission, age, and pre-stroke modified Rankin Scale.

## 3. Results

### 3.1. Study Population

**Figure 1:**
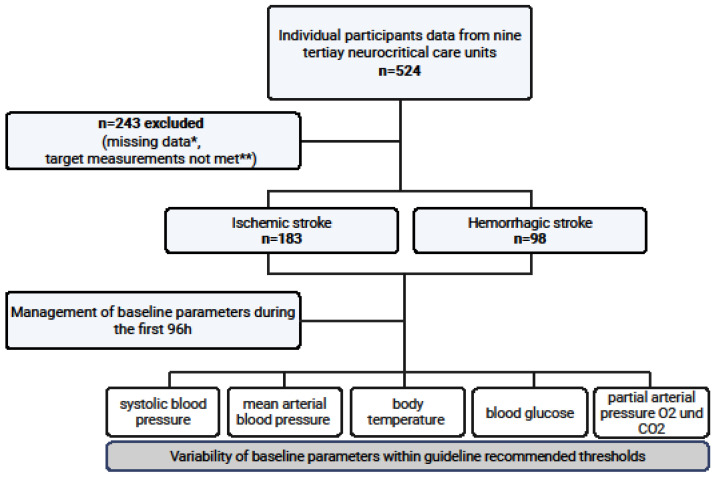
**Flow chart of study participants:** A total of 524 neurocritical care patients treated at nine tertiary University Hospitals in Germany were included over a 12-month period (1 January to 31 December 2021). Of these, 243 patients were excluded due to missing data * (n = 20) and because predefined criteria for target measurements were not met (n = 223) **. The final cohort comprised 281 patients, including 183 with ischemic stroke and 98 with hemorrhagic stroke. Clinical parameters, including systolic blood pressure, mean arterial blood pressure, body temperature, blood glucose levels, and partial arterial pressure of oxygen and carbon dioxide, were assessed at NICU admission and subsequently every 4 h for the initial 96 h of intensive care treatment. * Missing data were defined as incomplete documentation (>33% missing data) or insufficient availability of the 4 h measures during the first 96 h of treatment. ** Patients were excluded because ≤70% of all recorded physiological parameter values were within guideline-recommended ranges.

A total of 524 participants admitted to tertiary neurocritical care units were screened for eligibility (Figure 1). The final study cohort comprised 281 patients, including 183 patients with ischemic stroke (65.1%) and 98 patients with hemorrhagic stroke (34.9%). Table 1 presents the demographic and clinical baseline characteristics of the study population. The mean age of the participants was 69.0 years (IQR 60–78), with females accounting for 57.3%. At the time of admission, the median NIHSS score was 20 (IQR 12–34). Regarding treatment, intravenous thrombolysis was administered in 32.8% of ischemic stroke cases, and 68.3% received endovascular therapy. Among patients with hemorrhagic stroke, 32.0% underwent surgical hematoma evacuation. In terms of outcomes, the median NIHSS score at discharge was 20. The overall in-hospital mortality rate was 46.3%. All patients in this study were mechanically ventilated for at least 24 h during the observation period.

### 3.2. Variability of Physiological Parameters During the First 96 Hours

**Figure 2:**
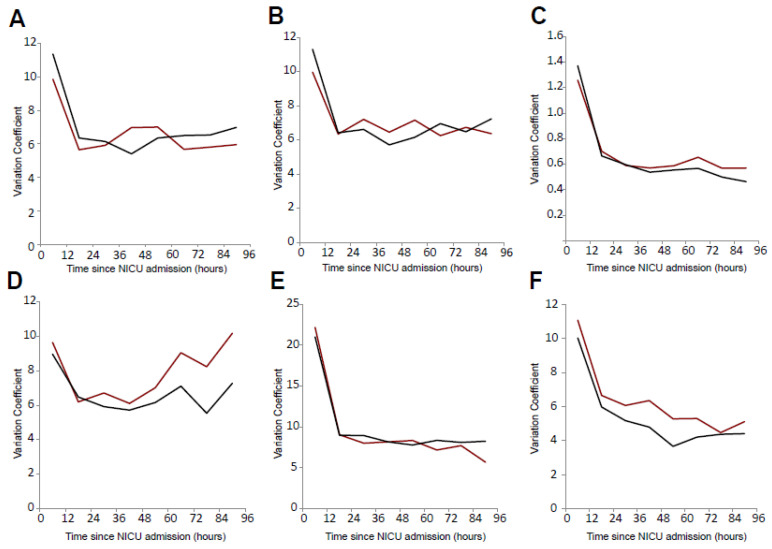
Variability of physiological parameters during the first 96 h after admission: the coefficient of variation (CV) was calculated in consecutive 12 h intervals over the first 96 h after admission for (**A**) systolic blood pressure, (**B**) mean arterial pressure, (**C**) body temperature, (**D**) blood glucose, (**E**) arterial partial pressure of oxygen (PaO_2_), and (**F**) arterial partial pressure of carbon dioxide (PaCO_2_). Analyses were performed separately for ischemic and hemorrhagic stroke; black lines represent ischemic stroke and red lines represent hemorrhagic stroke.

For all investigated parameters (systolic and diastolic blood pressure, mean arterial pressure (MAP), body temperature, blood glucose, PaO_2_ and PaCO_2_), we calculated the coefficient of variation in consecutive 12 h intervals over the first 96 h after stroke onset. Analyses were performed separately for ischemic and hemorrhagic stroke (Figure 2).

Across all parameters, the CV was highest within the first 24 h after stroke onset, irrespective of stroke subtype. This early peak was consistently observed in both ischemic and hemorrhagic stroke. After the initial 12–24 h, the CV stabilized and remained largely constant across subsequent intervals, with no relevant differences between ischemic and hemorrhagic stroke.

Blood glucose represented an exception to this overall pattern. After approximately 60 h, the CV of glucose increased again. This secondary rise was observed in both ischemic and hemorrhagic stroke, but it was more pronounced in patients with hemorrhagic stroke (CV glucose 60–72 h: [8.23]; 72–96 h: [10.18]). For all other parameters, the CV remained stable after the early phase, without evidence of a comparable secondary increase.

### 3.3. Association of Physiological Variability Within Guideline-Recommended Ranges with Clinical Outcomes

**Figure 3:**
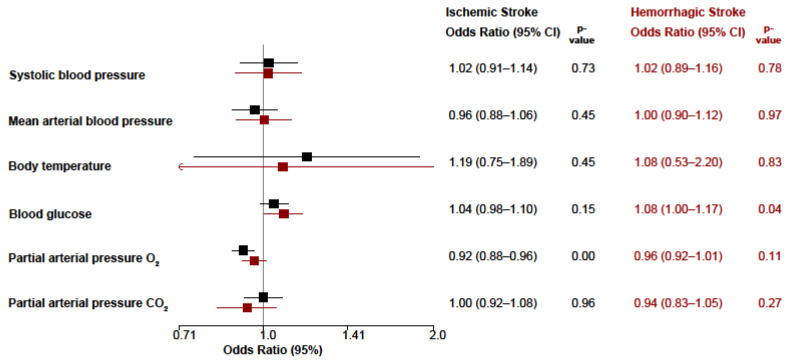
Association between variability of physiological parameters and in-hospital mortality: odds ratios (ORs) with 95% confidence intervals (CIs) for the association between the coefficient of variation (CV) of physiological parameters during the first 96 h of intensive care treatment and in-hospital mortality are shown, stratified by stroke subtype. Black symbols represent patients with ischemic stroke, and red symbols represent patients with hemorrhagic stroke. Physiological parameters include systolic blood pressure, mean arterial pressure, body temperature, blood glucose, arterial partial pressure of oxygen (PaO_2_), and arterial partial pressure of carbon dioxide (PaCO_2_).

In adjusted analyses, we assessed the association between physiological variability within guideline-recommended ranges of systolic blood pressure, mean arterial pressure, body temperature, blood glucose, PaO_2_, PaCO_2_ and clinical outcomes. All models were adjusted for age, baseline NIHSS score, and pre-stroke modified Rankin Scale and were operated separately for ischemic and hemorrhagic stroke (Figure 3).

No significant associations were observed between variability of physiological parameters within guideline-recommended ranges and clinical outcomes in patients with ischemic stroke. In patients with hemorrhagic stroke, blood glucose variability was associated with in-hospital mortality (adjusted OR 1.08; 95% CI, 1.00–1.17; *p* = 0.04), whereas no other significant associations were identified.

## 4. Discussion

This multicenter observational study represents the first investigation of variability in six key modifiable physiological parameters in neurocritical care patients with acute cerebrovascular diseases. Physiological variability peaked within 24 h after stroke onset, with blood glucose demonstrating a secondary increase in variability, predominantly in hemorrhagic stroke. Blood glucose revealed a modest association with in-hospital mortality, while variability of other parameters within guideline-recommended ranges was not associated with clinical outcomes.

The early peak in physiological variability observed across all parameters, despite values remaining within guideline-recommended ranges, likely reflects the hyperacute phase after stroke onset [21]. From a pathophysiological perspective, acute cerebrovascular injury is associated with transient autonomic dysfunction, including impaired baroreflex circuits, altered autonomic balance [22,23,24], and impaired cardiorespiratory drive, which can increase short-term fluctuations of cardiovascular and metabolic parameters even when absolute values remain within target ranges [25,26]. Acute neuroendocrine stress responses and early systemic inflammation, pain, agitation and sleep–wake disruption can amplify variability further [27,28]. Meanwhile, evolving cerebral edema and rising intracranial pressure may cause additional hemodynamic and respiratory instability [27,28,29].

In addition, care-related factors during the early phase may amplify within-range variability. Pre- and intra-hospital transport, handovers, diagnostic procedures, and urgent therapeutic interventions can introduce short-term fluctuations of physiological parameters that remain within guideline-recommended limits [30,31,32,33]. The early ‘settling’ effects after arrival, such as establishing reliable vascular access and arterial lines, calibrating monitors, initiating consistent sedation and analgesia, and standardizing ventilator and oxygen settings, may also reduce apparent variability by improving measurement accuracy and minimizing intermittent stressors [34,35,36,37]. After admission to the neurocritical care unit, continuous monitoring, protocolized management, and higher adherence to guideline-based targets may reduce such within-range fluctuations, contributing to the observed stabilization of variability after the first 24 h [38,39,40,41].

The distinct temporal pattern observed for blood glucose, characterized by a secondary increase in variability, is consistent with prior evidence highlighting the clinical relevance of dysglycemia in critically ill patients [42,43,44]. In the NICE-SUGAR trial, intensive glucose control was associated with increased mortality compared with conventional management, underscoring the potential harm of both hypoglycemia and excessive glucose fluctuations in critical illness [17,45]. Our findings extend these observations to a neurocritical care stroke population, suggesting that blood glucose variability may be particularly relevant in patients with hemorrhagic stroke rather than absolute glucose levels alone [46,47,48]. The secondary increase in glucose variability in this subgroup may reflect heightened systemic stress responses, intercurrent complications such as infection or renal dysfunction, or evolving nutritional and insulin management during prolonged critical care [49,50,51,52]. In addition, fluctuating glucose levels may contribute to secondary brain injury through mechanisms such as metabolic instability, oxidative stress, inflammatory activation, and impaired cellular energy homeostasis. In patients with hemorrhagic stroke, these effects may be particularly relevant given the vulnerability of perihematomal tissue and the frequent coexistence of systemic complications during neurocritical care. From a clinical perspective, our findings do not support overly aggressive glucose-lowering. However, they suggest that avoiding significant glucose fluctuations while maintaining guideline-recommended targets may be a clinically relevant management strategy. Further prospective studies are needed to determine whether strategies aimed at improving glycemic stability are associated with better outcomes in hemorrhagic stroke.

Notably, variability of other physiological parameters within guideline-recommended ranges was not associated with adverse clinical outcomes. This finding suggests that, once guideline thresholds are respected, residual within-range variability of blood pressure, temperature, and gas exchange may have limited prognostic relevance in the neurocritical care setting [53,54]. From a clinical perspective, our findings may indicate that maintaining physiological parameters within guideline-recommended targets is more relevant than minimizing small within-range fluctuations, particularly for blood pressure and temperature management. Accordingly, these data support a pragmatic focus on avoiding clinically relevant deviations beyond established target ranges rather than strict suppression of minor fluctuations within those ranges. These results support current guideline-based management strategies that prioritize avoidance of extreme values rather than strict minimization of physiological variability [3,16,37].

Several limitations warrant consideration. The observational design precludes causal inference, and residual confounding cannot be excluded despite multivariable adjustment. Variability metrics were derived from routinely collected clinical data and may have been influenced by measurement frequency and clinical decision-making. Moreover, inclusion was limited to patients with a minimum NICU stay of 4 days, in line with the predefined 96 h observation window and 4 h interval recordings used for variability assessment. Although this criterion improved completeness and comparability of serial physiological measurements across centers, it may also have introduced selection bias by excluding patients with shorter NICU stays, particularly those with less severe disease courses or earlier transfer from neurocritical care. In addition, more detailed baseline demographic characteristics, vascular risk factors, and subtype-specific markers of disease burden and complications (e.g., vasospasm or pneumonia) were not available in a sufficiently standardized and complete manner across participating centers due to the retrospective study design, thereby limiting more granular analyses of the individual stroke subtypes. Although the multivariable models were adjusted for age, baseline NIHSS score, and pre-stroke mRS, additional potentially relevant confounders such as vascular risk factors, stroke burden, recanalization status, vasospasm, and other neurocritical care complications could not be included because these data were not available in a sufficiently complete and standardized manner across centers. Consequently, residual confounding cannot be excluded, and the findings should be interpreted with appropriate caution. A further limitation is that ICH and SAH, although distinct pathophysiological entities, were combined in the variability and outcome analyses under the category of hemorrhagic stroke. This approach was chosen to preserve statistical reliability and avoid overstratification, but it may have reduced the resolution of specific diseases. Furthermore, the study population consisted exclusively of mechanically ventilated patients treated at tertiary centers, which may limit generalizability to less severely affected stroke populations.

## 5. Conclusions

In conclusion, physiological variability within guideline-recommended ranges appears to be largely confined to the early phase after stroke onset and shows limited associations with short-term clinical outcomes. The association observed for blood glucose variability in hemorrhagic stroke warrants further investigation in prospective studies. Due to the retrospective design and lack of standardized long-term outcome measures, future prospective studies should assess the association between early variability in physiological parameters and long-term functional outcomes.

## Figures and Tables

**Table 1:** Characteristics and outcome parameters of the overall cohort stratified by ischemic and hemorrhagic stroke.

Baseline Characteristics	Ischemic Stroke (n = 183)	Hemorrhagic Stroke (n = 98)	
		ICH (n = 75)	SAH (n = 23)
Age, ^a^ years	70 (14.3)	65 (14.3)	62 (14.5)
Sex, ^b^ female	106 (57.9)	44 (58.7)	11 (47.8)
**Admission status**			
NIHSS at admission (0–42) ^c^	17 (12–27)	18 (17–36)	34 (4–36)
Pre-mRS ^c^	0 (0–2)	0 (0–1)	0 (0–1)
Preclinical intubation ^b^	134 (73.2)	58 (77.3)	20 (87.0)
**Disease-specific interventions**			
EVT (ischemic stroke) ^b^	125 (68.3)		
IVT (ischemic stroke) ^b^	60 (32.8)		
Decompressive craniectomy (ischemic stroke) ^b^	7 (3.8)		
Surgical hematoma evacuation (hemorrhagic stroke) ^b^		25 (33.3)	
Coiling (hemorrhagic stroke) ^b^			23 (87.0)
Clipping (hemorrhagic stroke) ^b^			4 (17.4)
EVD (hemorrhagic stroke) ^b^		48 (64.0)	23 (95.7)
Lumbar drain (hemorrhagic stroke) ^b^		13 (17.3)	11 (47.8)
**Outcome parameter**			
NIHSS at discharge ^c^	16 (9–24)	27 (16–38)	21 (20–33)
In-hospital mortality ^b^	81 (44.3)	35 (46.1)	14 (60.9)
Duration of ventilation, hours ^c^	216 (35–365)	263 (195–369)	363 (273–485)

^a^ mean ± SD; ^b^ n (%); ^c^ median (interquartile range: 25th–75th percentile). Abbreviations: EVD, external ventricular drain; EVT, endovascular therapy; IVT, intravenous thrombolysis; ICH, intracerebral hemorrhage; mRS, modified Rankin Scale (0 meaning no deficit to 6 meaning death); NIHSS, National Institutes of Health Stroke Scale (ranging from 0 meaning no deficit to −40 meaning severe neurological deficit; 40 is the maximum because comatose ataxia is not scored), applied for patients with ischemic stroke and intracerebral hemorrhage; SAH, subarachnoid hemorrhage.

## Data Availability

The datasets used and/or analyzed during the current study are available from the corresponding author upon reasonable request.
